# Rehabilitation of Eyelid Malpositions Secondary to Facial Palsy

**DOI:** 10.4274/tjo.13549

**Published:** 2017-06-01

**Authors:** Şeyda Karadeniz Uğurlu, Mustafa Karakaş

**Affiliations:** 1 İzmir Katip Çelebi University Faculty of Medicine, Department of Ophthalmology, İzmir, Turkey; 2 Malatya State Hospital, Ophthalmology Clinic, Malatya, Turkey

**Keywords:** Facial paralysis, eyelid malposition, lagophthalmos, gold weight, suborbicularis oculi fat pad

## Abstract

**Objectives:**

To evaluate patient satisfaction and outcomes of surgical treatment of eyelid malpositions secondary to facial palsy.

**Materials and Methods:**

Consecutive patients with facial palsy who underwent surgical treatment by the same surgeon at İzmir Katip Çelebi University Atatürk Training and Research Hospital between Jan 2007 and Dec 2012 were included in the study. Ophthalmic examination findings, surgical approaches, and their outcomes were evaluated. A successful result for upper eyelid position was defined as more than 50% reduction in lagophthalmos and induction of less than 2 mm of ptosis. A successful outcome for lower eyelid position was defined as the lower eyelid residing at or within 1 mm above or below the limbus. Linear visual analog scale 1 (VAS-1) (subjective complaints) and VAS-2 (cosmetic outcome), both ranging from 0 to 10, were used to compare preoperative findings with findings at last postoperative visit.

**Results:**

The mean age of the 14 female and 21 male patients was 54.5±19.9 years. Gold weight implantation (n=31), lateral tarsal strip (n=22), tarsorrhaphy (n=15), suborbicularis oculi fat elevation (n=16), hard palate graft (n=14), and eyebrow ptosis repair (n=6) were performed. Average follow-up time was 17.9±16.9 months (range, 2-60). Surgical success rates were 90% for upper lids and 75% for lower lids. Mean lagophthalmos decreased from 7.1±2.7 mm to 1.6±1.6 mm postoperatively (p=0.000). The use of lubricating drops and gels was reduced from average preoperative daily values of 5.3±2.5 drops and 1.3±0.6 gel applications to 4.4±1.4 and 0.6±0.6, respectively (p=0.003, p=0.001).

**Conclusion:**

An individualized surgical approach tailored according to each patient’s severity of facial palsy and associated malpositions resulted in both functional and aesthetic improvements in our patients.

## INTRODUCTION

In order to protect the functional and structural integrity of the eye following facial palsy, it is of utmost importance to accurately assess and plan appropriate therapeutic approaches to both ocular surface problems and eyelid malpositions. Inability of the eyelids to close completely can lead to a progressive continuum of problems ranging from diminished or absent blinking reflex and exposure keratopathy to corneal ulceration, perforation, and even blindness.^1^ Epiphora, lid retraction, paralytic ectropion, and cosmetic issues may also occur.

The most common form of facial nerve palsy is idiopathic facial paralysis.^2^ Infectious agents, trauma, neoplasmas, and autoimmune diseases are other disorders implicated in its etiology. While lagophthalmos secondary to idiopathic facial paralysis may be reversible, most other causes of facial nerve palsy result in irreversible lagophthalmos.

Various medical and surgical approaches are used to treat eyelid malpositions and ocular surface problems secondary to facial palsy. The primary goal of medical therapy is to ensure ocular surface integrity and patient comfort. Individual differences like disease severity and the patient’s age and expectations play as much a role in the planning of surgical interventions for malpositions as eyelid anatomy and physiology.

In this study we aimed to evaluate the characteristics and surgical outcomes of patients treated for eyelid malposition secondary to facial palsy in the Clinic of Ophthalmology, İzmir Katip Çelebi University Atatürk Training and Research Hospital between January 2007 and December 2013.

## MATERIALS AND METHODS

After receiving approval from the İzmir Katip Çelebi University Atatürk Training and Research Hospital Ethics Committee, the medical records of patients who underwent surgical treatment by the same surgeon for eyelid malposition secondary to facial palsy in the oculoplastic surgery division between January 2007 and December 2013 were analyzed retrospectively. The patients’ demographic characteristics, etiology of facial palsy, visual acuity, and anterior and posterior segment examination findings were recorded. Exposure keratopathy was graded in 5 levels based on the degree of corneal involvement (grade 0: none, grade 1: mild superficial punctate keratopathy, grade 2: punctate keratopathy on the inferior quarter of the cornea, grade 3: punctate keratopathy on the inferior third to half of the cornea, grade 4: punctate keratopathy on more than half of the cornea or any erosion or ulceration).^3^ Patients were evaluated for the presence of Bell’s phenomenon, any other pathologies limiting lower and upper lid movement, and degree of lagophthalmos.

Surgical methods utilized included gold weight implantation, lateral tarsal strip (LTS), suborbicularis oculi fat pad (SOOF) elevation with subperiosteal approach, hard palate graft, and tarsorrhaphy. A suitable combination of procedures was determined for each patient based on amount of lagophthalmos, position of the lower lid/cheek complex, and the patient’s preference. In gold weight implantation procedures, the implant weight was determined using trial weight sets. The weight yielding the best lagophthalmos correction and minimal (approximately 1 mm) ptosis was selected.

To evaluate lid symmetry postoperatively, margin reflex distance-1 (MRD1) (distance from upper lid margin to light reflex) and MRD2 (distance from lower lid margin to light reflex) were recorded. Surgical success of upper eyelid procedures was defined as at least 50% reduction in lagophthalmos and induction of less than 2 mm of ptosis. For lower eyelids, surgical outcomes were defined as favorable when the lower lid rested at the limbus, acceptable when the lower lid margin rested within 1 mm above or below the lower limbus, and unsatisfactory when the lower lid rested more than 1 mm above or below the limbus. Patients with good or acceptable lower lid position were considered successful.

The visual analog scale (VAS) was used to compare preoperative findings with findings at the final postoperative follow-up examination. Two different scales evaluating subjective complaints (VAS-1) and cosmetic outcome (VAS-2) were applied. Patients were asked to select the most appropriate value that represented their outcome from a scale of 0 to 10. VAS-1 scoring assessed pre- to postoperative reduction in symptoms as 0: no reduction, 5: some reduction, 10: my symptoms are completely gone; the VAS-2 evaluated postoperative cosmetic appearance as 0: no change, 5: some improvement, I am satisfied, 10: big improvement, I am very satisfied.

In addition, the patients’ artificial tear drops/gel application frequency was assessed pre- and postoperatively.

## RESULTS

Thirty-six eyes of 35 patients (21 male, 14 female) were included in the study. The patients’ ages ranged from 11 to 93, with a mean of 54.5±19.9 years. Facial palsy affected the right side in 18 patients and the left side in 16 patients. One patient with bilateral facial palsy following trauma underwent procedures on both sides. Data from this patient were not included in analyses comparing the operated eyes and fellow eyes.

Intracranial tumor surgery was the most common cause of facial palsy; the other causes are listed by order of frequency in Table 1.

Time between onset of facial palsy and surgery ranged from 0 to 69 years, with a mean elapsed time of 14.2±20.6 years. Mean postoperative follow-up time was 17.9±16.9 (range, 2-60) months.

Mean visual acuity assessed by Snellen chart was 0.60±0.34 preoperatively and 0.64±0.32 postoperatively (p=0.078). The mean frequency of lubricant eye drop application pre- and postoperatively was 5.33±2.47 and 4.38±1.36 drops daily, respectively (p=0.03); for gel formulations, the mean frequency was 1.35±0.6 applications preoperatively and 0.56±0.71 postoperatively (p=0.001).

Pre- and postoperative mean keratopathy grades were 2.11±1.45 and 0.92±1.23, respectively (p=0.000).

Several procedures were utilized, the most common being gold weight implantation. The surgical procedures performed are presented in Table 2.

**Figure 1:**
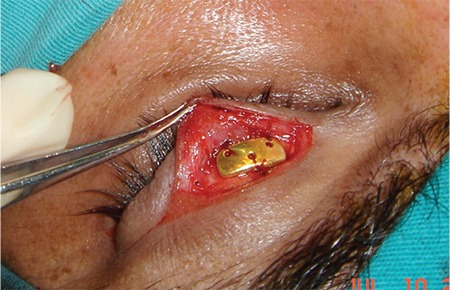
The gold weight implant was secured to the tarsal surface with 3 sutures

Thirty-one patients underwent gold weight implantation, and the average implant weight was 1.19±0.28 g. The implants ranged in weight from 0.6 to 1.6 g (Figure 1). The success rate among these patients was 90%. A total of 3 patients did not meet the criteria for successful outcome: less than 50% reduction in lagophthalmos was achieved in 1 patient and greater than 2 mm ptosis was induced in 2 patients.

Mean amount of lagophthalmos decreased from 7.08±2.7 mm to 1.61±1.57 mm postoperatively (p=0.000; Table 3). Preoperative and postoperative amount of lagophthalmos was classified as 0-3 mm, 4-6 mm, or ≥7 mm; 22 patients (61.1%) had ≥7 mm lagophthalmos preoperatively. There were no patients with lagophthalmos ≥7 mm in the postoperative period (Table 4).

Postoperative MRD1 values were 2.06±1.12 mm on the operated side and 2.97±0.59 mm on the unoperated side. MRD1 differed significantly between operated and fellow eyes (p=0.003; Table 5). The distance from the lower lid to the limbus was 0.94±0.68 mm on the operated side and 0.47±0.62 mm on the unoperated side (p=0.067). Mean MRD2 values were 6.44±0.68 mm on the operated side and 5.97±0.62 mm on the unoperated side. The differences in lower lid-to-limbus distance and MRD2 values between operated and unoperated eyes were not significant (p=0.067; Table 5).

**Figure 2:**
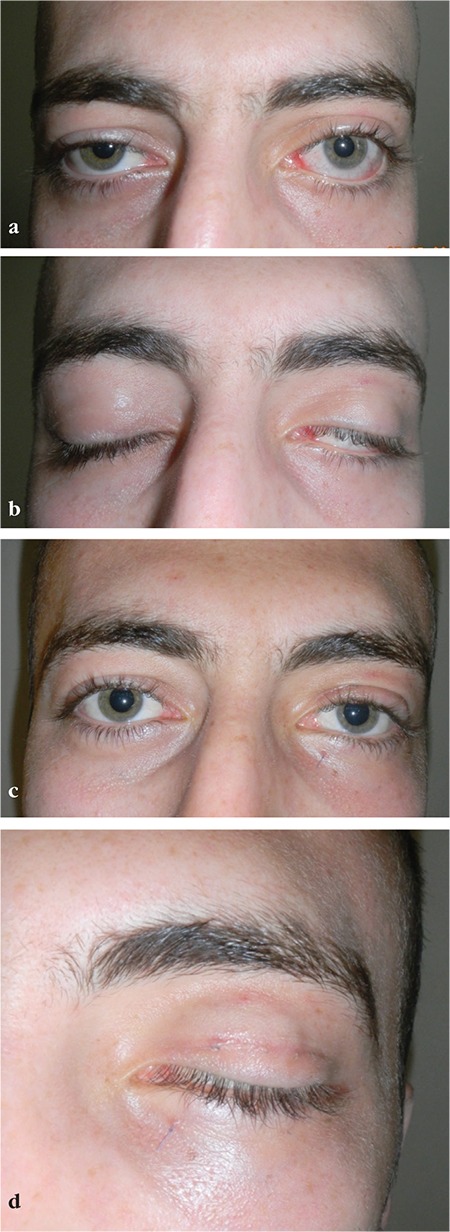
a, b) Upper-lower lid retraction and lagophthalmos due to left facial palsy. c, d) Improved upper and lower lid position and marked reduction in lagophthalmos were apparent after gold weight implantation, suborbicularis oculi fat elevation, hard palate graft, and lateral tarsal strip procedures

**Figure 3:**
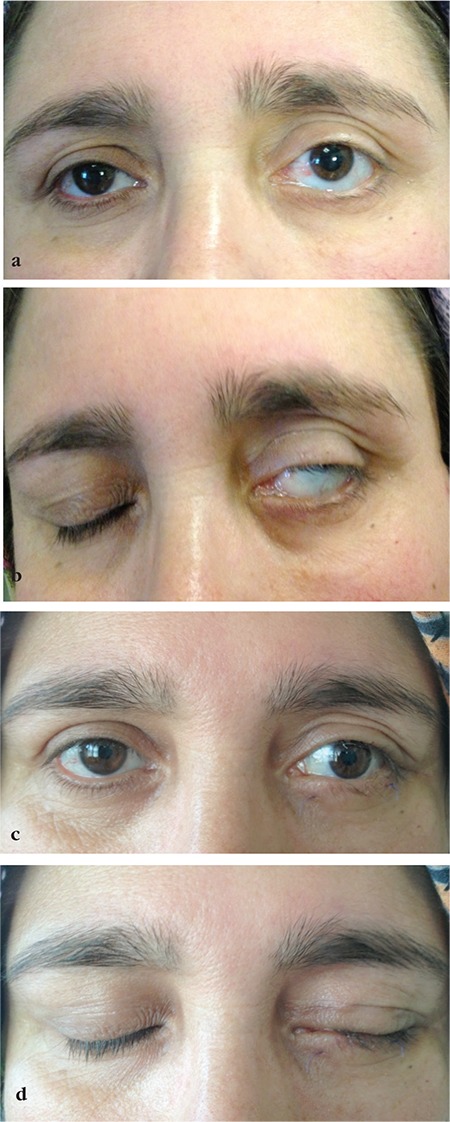
a, b) Mild upper lid retraction, pronounced lower lid retraction, and lagophthalmos due to left facial palsy. c, d) Lagophthalmos was alleviated and the lower lid rested at the limbus after gold weight implantation, suborbicularis oculi fat elevation, hard palate graft, and lateral tarsal strip procedures

According to the criteria for lower lid success, of the 16 eyes of 15 patients who underwent SOOF elevation (LTS and/or hard palate graft), surgical success was achieved in 75% (6 successful, 6 partially successful) (Figures 2 and 3), while the other 25% (n=4) were not considered successful due to more than 1 mm of retraction.

After surgery, the patients’ mean scores for subjective complaints (VAS-1) and cosmetic appearance (VAS-2) fell by 6.17±1.3 and 6.04±1.99, respectively.

Implant migration or expulsion was not observed in any of the patients in the early or late postoperative period. One patient with gold weight implant complained of increased heat and localized redness on the first postoperative day. The patient’s findings resolved with oral antibiotic therapy. No other postoperative local or systemic complications were noted following the other surgical procedures.

## DISCUSSION

The etiology of facial palsy includes idiopathic, traumatic, infectious, and neoplastic causes. May and Klein^4^ determined that idiopathic facial paralysis was the most common cause (49-51%). Studies conducted in Turkey have reported a comparable distribution.^5,6^ In the present study, we found that facial paralysis resulting from surgical trauma and idiopathic paralysis were the first and second most common causes.

In facial palsy, visual acuity may decline due to exposure keratopathy and the toxic effects of applied medical therapies on the cornea. We did not observe a statistically significant change in visual acuity after surgery in the present study. Similarly, Berghaus et al.^7^ noted no significant difference between pre- and postoperative visual acuity levels.

The reduced tear production, increased evaporation, and disruptions in tear film stability and the pump mechanism of the lacrimal drainage system that occur in facial palsy may give rise to ocular surface disorders.^1^ Numerous studies have reported regression of ocular surface problems and keratopathy findings following both medical and surgical therapies.^7,8,9,10^ Amer et al.^3^ performed two forms of gold weight implantation and applied the same keratopathy classification system that we utilized in the present study; they reported that intervention reduced keratopathy severity from a preoperative average of 1.2-1.4 to a postoperative average of 0.3-0.4. Similarly, the mean keratopathy severity in our study was 2.11±1.45 preoperatively and regressed to 0.92±1.23 postoperatively. These values also demonstrate that the patients in our study had more severe keratopathy initially and treatment provided significant improvement.

In facial palsy patients, surgical interventions to correct lid malpositions are performed to reduce exposure keratopathy. It can be expected that improvement in keratopathy will reduce the need for patients to use topical lubricants. We observed a significant pre- to postoperative reduction in the number of daily applications of both drop and gel forms of topical lubricants. Seiff et al.^11^ also reported a reduction in topical lubricant use in their study including 12 patients. Golio et al.^8^ determined in their study that all of the 44 patients who used a lubricant preoperatively reduced their usage postoperatively.

Many different methods may be utilized in the treatment of lid malpositions due to facial palsy. In the present study, the patients were not randomized; the surgical plan was determined based on patients’ degree of paralysis and the position of the upper lid and lower lid/cheek complex. Tarsorrhaphy alone was preferred in a small minority of patients. This preference was based both on the fact that the procedure was not pleasing cosmetically and that none of the patients had accompanying fifth cranial nerve involvement. Methods like levator recession or müllerectomy were not preferred for upper lid retraction; instead, we opted for gold weight implantation, a procedure easy to perform and readily adjusted to meet each patient’s needs. For lower lid malpositions, combined procedures yielded the best results. In patients with pronounced lower lid retraction, we used the LTS procedure to establish lateral canthal support in addition to SOOF elevation and hard palate graft to effectively lift atonic lids. Eyebrow surgeries were planned for patients with upper visual field loss or cosmetic concerns as the last step of rehabilitation, after surgeries that protect the cornea and correct eyelid malposition.

The preoperative amount of lagophthalmos caused by facial palsy varies, often ranging from 4 to 8 mm.^5,6,7^ In our study, the mean preoperative amount of lagophthalmos was 7 mm; compared to other reports in the literature, our study group included patients with more severe lagophthalmos.

Gold weight implantation is often used in the treatment of lagophthalmos due to upper lid retraction because the implant is relatively inert, is well tolerated by patients, yields favorable cosmetic outcomes, and the procedure is reversible. We performed gold weight implantation in 31 patients with a mean implant weight of 1.19±0.28 g and encountered no serious complications. Comparable mean implant weight values have been reported in previous studies.^10,11,12^

Townsend^10^ performed gold weight implantation in 23 patients and reduced the amount of lagophthalmos from a preoperative mean of 4 mm to a postoperative mean of 0.5 mm. Berghaus et al.^7^ reported the mean lagophthalmos amount as 5 mm preoperatively and 0.3 mm postoperatively in 33 patients that underwent gold weight implantation. Akçay et al.^5^ performed gold weight implantation in 18 patients and reported that the preoperative mean lagophthalmos amount of 4.96 mm decreased to 1.6 mm postoperatively. In our study, gold weight implantation performed alone (n=3) or in combination with other procedures (n=28) reduced the amount of lagophthalmos from 7.1 mm to 1.6 mm.

Aggarwal et al.12 performed gold weight implantation in 29 patients and accepted outcomes with at least 50% reduction in lagophthalmos with induction of less than 2 mm ptosis as successful. The mean amount of lagophthalmos in their study fell from 7 mm to 2.3 mm, and their success rate was 68.9%. In the present study, we achieved a success rate of 90% among the 31 patients we treated with gold weight implantation. Three of our patients did not meet the criteria for successful outcome due to less than 50% reduction in lagophthalmos in 1 patient and induction of more than 2 mm ptosis in 2 patients.

SOOF elevation is an effective method for correcting lower lid/cheek problems due to seventh nerve palsy. It has been successfully used to correct eyelid asymmetry and achieve a better lower lid position.^13,14,15^ SOOF elevation was shown to be particularly effective in congenital cases.^15^ Ben Simon et al.^16^ reported that successful results were achieved with subperiosteal midface lift performed in 34 patients with lower lid retraction (6 of whom had facial palsy) and that the patients whose procedure included a hard palate graft had greater improvement in MRD2.^16^ In our study we performed SOOF elevation in 16 eyes of 15 patients. The procedure was combined with LTS and/or hard palate graft. Therefore, we are unable to comment on the effectiveness of SOOF elevation alone, but our study demonstrated that elevation of the lower lid/cheek complex as a whole provided significant improvement in patients with severe lower lid retraction.

Harvesting a hard palate graft causes temporary discomfort, and healing of the donation site usually takes time. However, previous studies have shown that procedures addressing multiple elements of lower lid retraction yield more favorable results.^17^ Wearne et al.^18^ reported good or acceptable outcomes in 85% and unsatisfactory outcomes in 15% of their study of 102 eyes of 68 patients with lower lid malpositions corrected using autogenic hard palate mucosa. Of the 16 eyes of 15 patients that underwent lower lid surgery in our study, we achieved good or acceptable results in 75% and unsatisfactory results in the remaining 25%. However, the patient group in Wearne et al.’s^18^ study included only 3 patients with paralytic ectropion, and in most patients, hard palate graft was performed to correct lower lid retraction secondary to thyroid-associated orbitopathy. Furthermore, time elapsed since the procedure is also a determinant of lower lid position, as retraction may increase with longer follow-up. Wearne et al.^18^ evaluated their patients at 3 months postoperatively, whereas we evaluated lid position based on findings at the patients’ final examination after a mean postoperative follow-up period 17.9±16.9 months.

The LTS procedure preserves the natural anatomy while effectively correcting horizontal laxity. Chang and Olver^9^ performed an augmented LTS procedure in 14 patients with paralytic lagophthalmos and determined their mean postoperative MRD2 to be 5 mm. The authors reported an average change in MRD2 of 3 mm. In a study by Loyo et al.,^19^ 37% of 47 patients that had surgery for paralytic lower lid retraction benefited from standard LTS. We also employed the standard LTS procedure but based on lower lid/cheek position and patient preference, we preferred LTS for mild cases and SOOF + hard palate graft + LTS for more severe cases. Comparison with MRD2 values of patients’ non-paralytic eyes showed that patients in both groups had good lower lid symmetry postoperatively.

Various scales are employed to evaluate patients’ perceptions of their surgical outcomes after undergoing procedures for facial palsy. Sönmez et al.^20^ used the visual analogue scale (VAS) to evaluate postoperative changes in ocular symptoms in 41 patients that underwent gold weight implantation. Patients showed the highest satisfaction in terms of eye closure ability, while visual acuity received the lowest score. We utilized the VAS-1 to assess postoperative changes in ocular complaints and the VAS-2 to assess postoperative cosmetic changes, and obtained results similar to those of Sönmez et al.^20^ These results indicated that surgical therapy provided significant improvement in the ocular complaints of the patients, and in their subjective cosmetic perceptions.

Ensuring regular follow-up is important for patients undergoing surgical treatment for facial palsy. Over time, patients with gold weight implants may experience implant superficialization, ptosis, residual lagophthalmos, increased upper lid retraction, or implant expulsion, and may require additional surgery.^19,21^ The same applies for procedures on the lower lid; recurrence of lower lid retraction in the long term may occur, resulting in reduced efficacy of the procedure and unfavorable cosmetic changes.^16^ The mean postoperative follow-up time in our study was 17.9±16.9, ranging from 2-60 months. Other than signs of infection during early follow-up in one patient, we observed no other serious complications in our patients.

## CONCLUSION

This retrospective study evaluating the treatment outcomes of eyelid malpositions secondary to facial palsy cannot compare and determine the superiority of the different surgical procedures over one another. Prospective studies utilizing classification systems focused on ocular findings may be more informative in this respect. On the other hand, our study demonstrates that satisfying outcomes can be achieved using an individualized approach in which treatment options are determined based on the patient’s clinical presentation. This approach provides a high rate of improvement in both ocular and cosmetic complaints. Even with successful surgical outcomes, long-term follow-up is necessary for patients with facial palsy in order to detect and manage lid position changes that may occur over time and to enable timely intervention for the subsequent ocular surface problems that may arise.

## Figures and Tables

**Table 1:**
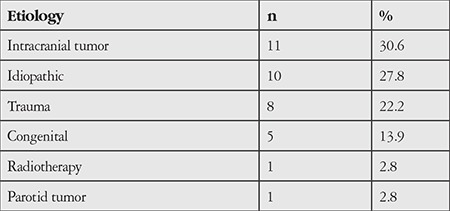
Distribution of patients by facial palsy etiology

**Table 2:**
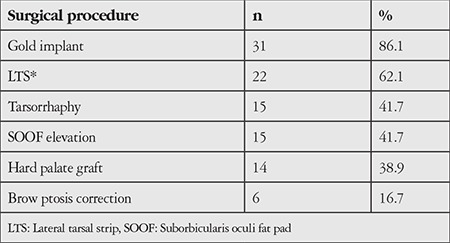
Distribution of surgical procedures

**Table 3:**
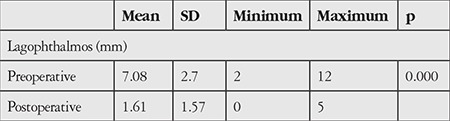
Comparison of pre- and postoperative lagophthalmos amounts

**Table 4:**
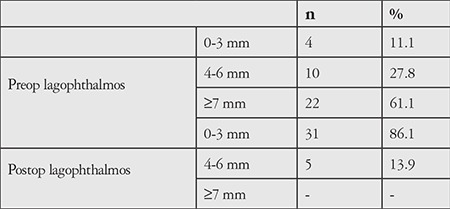
Pre- and postoperative classification of degree of lagophthalmos

**Table 5:**
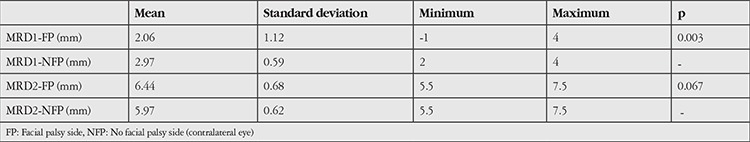
Postoperative MRD1 and MRD2 values

## References

[1] Rahman I, Sadiq SA (2007). Ophthalmic management of facial nerve palsy: a review. Surv Ophthalmol..

[2] Bergeron CM, Moe KS (2008). The evaluation and treatment of upper eyelid paralysis. Facial Plast Surg..

[3] Amer TA, El-Minawi HM, El-Shazly MI (2011). Low-level versus high-level placement of gold plates in the upper eyelid in patients with facial palsy. Clin Ophthalmol..

[4] May M, Klein SR (1991). Differential diagnosis of facial nerve palsy. Otolaryngol Clin North Am..

[5] Akçay L, Kartal B, Doğan ÖK (2008). Fasiyal Paraliziye Bağlı Lagoftalmi Varlığında Üst Kapağa Altın Ağırlık Uygulaması ve Göz İçi Basıncına Etkisi. Turk J Ophthalmol..

[6] Yalaz M, Özcan AA, Demircan N, Yağmur M (2001). Fasiyal paralizili olgularda lagoftalminin düzeltilmesi. Turk J Ophthalmol..

[7] Berghaus A, Neumann K, Schrom T (2003). The platinum chain: a new upper-lid implant for facial palsy. Arch Facial Plast Surg..

[8] Golio D, De Martelaere S, Anderson J, Esmaeli B (2007). Outcomes of periocular reconstruction for facial nerve paralysis in cancer patients. Plast Reconstr Surg..

[9] Chang L, Olver J (2006). A useful augmented lateral tarsal strip tarsorrhaphy for paralytic ectropion. Ophthalmology..

[10] Townsend DJ (1992). Eyelid reanimation for the treatment of paralytic lagophthalmos: historical perspectives and current applications of the gold weight implant. Ophthal Plast Reconstr Surg..

[11] Seiff SR, Boerner M, Carter SR (1995). Treatment of facial palsies with external eyelid weights. Am J Ophthalmol..

[12] Aggarwal E, Naik MN, Honavar SG (2007). Effectiveness of the gold weight trial procedure in predicting the ideal weight for lid loading in facial palsy: a prospective study. Am J Ophthalmol..

[13] Chung JE, Yen MT (2007). Midface lifting as an adjunct procedure in ectropion repair. Ann Plast Surg..

[14] Graziani C, Panico C, Botti G, Collin RJ (2011). Subperiosteal midface lift: its role in static lower eyelid reconstruction after chronic facial nerve palsy. Orbit..

[15] Olver JM (2000). Raising the suborbicularis oculi fat (SOOF): its role in chronic facial palsy. Br J Ophthalmol..

[16] Ben Simon GJ, Lee S, Schwarcz RM, McCann JD, Goldberg RA (2006). Subperiosteal midface lift with or without a hard palate mucosal graft for correction of lower eyelid retraction. Ophthalmology..

[17] Tan J, Olver J, Wright M, Maini R, Neoh C, Dickinson AJ (2004). The use of porous polyethylene (Medpor) lower eyelid spacers in lid heightening and stabilisation. Br J Ophthalmol..

[18] Wearne MJ, Sandy C, Rose GE, Pitts J, Collin JR (2001). Autogenous hard palate mucosa: the ideal lower eyelid spacer? Br J Ophthalmol.

[19] Loyo M, Jones D, Lee LN, Collar RM, Molendijk J, Boahene KD, Ishii LE, Byrne PJ (2015). Treatment of the periocular complex in paralytic lagophthalmos. Ann Otol Rhinol Laryngol..

[20] Sönmez A, Oztürk N, Durmuş N, Bayramiçli M, Numanoğlu A (2008). Patients’ perspectives on the ocular symptoms of facial paralysis after gold weight implantation. J Plast Reconstr Aesthet Surg..

[21] Bladen JC, Norris JH, Malhotra R (2012). Indications and outcomes for revision of gold weight implants in upper eyelid loading. Br J Ophthalmol..

